# Exploring the Influence of Perceived Social Support on Medication Adherence Among Patients With Chronic Hypertension in 2024: A Cross-Sectional Study From Jeddah, Kingdom of Saudi Arabia

**DOI:** 10.7759/cureus.69522

**Published:** 2024-09-16

**Authors:** Sameer A Almaghamsi, Khalid M Alzahrani

**Affiliations:** 1 Preventive Medicine, Ministry of Health, Jeddah, SAU; 2 Preventive Medicine, Public Health Administration, Health Programs, Jeddah, SAU

**Keywords:** hypertension, medication adherence, medication non-adherence, perceived social support, primary healthcare

## Abstract

Background

There is no doubt that hypertension is one of the most widespread global health problems, and it is essential to comply with prescribed medications for optimal control. A lack of studies on the effects of perceived social support (PSS) on medication adherence among hypertension patients, especially in Jeddah, Saudi Arabia, made us conduct this study.

Methods

This was a cross-sectional study that targeted hypertension patients who were visiting primary healthcare centers under the Ministry of Health in Jeddah, Saudi Arabia. The Morisky Green and Levine (MGL) adherence scale and the Multidimensional Scale of Perceived Social Support (MSPSS) were used to assess adherence and perceived social support, respectively.

Results

The study included 377 hypertensive patients with a mean age of 57 years (SD = 10). The majority were male (n=226, 59.9%) and married (n=261, 69.2%). Educational levels varied, with 148 (39.3%) completing high school and 79 (21%) holding a bachelor’s degree or higher. Income distribution showed 166 (44%) earning less than 5,000 SAR and 47 (12.5%) earning over 15,000 SAR. Medication adherence, assessed using the MGL scale, revealed that 121 (32.1%) had low adherence, 206 (54.6%) had moderate adherence, and 50 (13.3%) had high adherence. Perceived social support, measured using the MSPSS scale, indicated that 219 (58.1%) reported high levels of support. A significant association between medication adherence and perceived social support was found (F = 10.293, p < 0.01), with moderate adherers having higher social support scores. Socio-demographic factors influencing adherence included marital status, education level, income, and occupation, with significant associations for each (p < 0.01). Married participants and those with higher education and income levels had greater adherence. Government employees showed the highest moderate adherence while the unemployed and homemakers had lower adherence. No significant associations were found between gender or age and adherence.

Conclusion

Higher levels of perceived social support, in addition to the influence of socio-demographic factors, are associated with better medication adherence. Targeted interventions addressing these factors could enhance medication adherence and improve health outcomes in this population.

## Introduction

Hypertension is the most prevalent noncommunicable disease (NCD) in primary care, with nearly 1.3 billion cases worldwide and predicted to rise to 1.5 billion by 2025 [1-3]. As the population ages and lifestyle risk factors like unhealthy diets increase, hypertension is becoming one of the leading risk factors for death and disability in the Kingdom of Saudi Arabia (KSA) [4,5]. Managing blood pressure is a significant challenge for healthcare providers due to the burden it imposes on the healthcare system, and sustainable social support is an important attribute of socioeconomic factors affecting treatment adherence. Social support encompasses emotional, informational, and practical support that may be received or perceived as available when needed from significant others, such as friends, coworkers, or family members, and access to adequate social support has been shown to improve treatment adherence and health outcomes for patients with chronic diseases [6,7]. Support encompasses a wide range of constructs, encompassing perceptions of support and receiving of support. Perceived social support refers to individuals’ perceptions of the level and quality of available social support and is considered more important than received social support [8-10].

Medication adherence refers to a patient's performance in accordance with their healthcare provider's advice [11]. In the United States (US), it was shown that about a third of patients suffer from uncontrolled hypertension, and medication non-adherence is a significant contributing factor [12]. Antihypertensive medication non-adherence is associated with an increased risk of adverse cardiovascular and cerebrovascular events, hospitalizations, and mortality [13,14].

The relationship between social support and treatment adherence is also moderated by several factors, such as the characteristics of the disease, the care regimen, and the patient's characteristics [15]. An empirical study in India found a significant positive correlation between social support and adherence, with a significant proportion of the variation in adherence between hypertensive cases attributed to social support [16]. Similarly, a Nigerian study concluded that spousal social support positively impacted medication adherence in Nigeria [17]. Studies in the US showed that social support was associated with a high level of medication adherence and lower odds of high blood pressure in patients [18,19]. On the other hand, a previous meta-analysis found that structural social support is not significantly related to overall adherence levels, but rather, adherence was significantly and positively related to the level of functional social support [20].

While in Saudi Arabia, the previous study showed that poor adherence was associated with lower overall perceived quality of life (QOL) and health [21], there have been no studies in Saudi Arabia that have examined the association between perceived social support and medication adherence. This study aims to fill the gap by studying hypertensive patients in primary health care centers in Jeddah, and exploring the role that perceived social support can play in medication adherence among hypertensive patients. This research could contribute to the development of health policy and public health initiatives aimed at improving the adherence of hypertensive patients in Saudi Arabia to their medication regimens.

## Materials and methods

Study design and settings

This is a cross-sectional study carried out in Jeddah, the commercial capital of Saudi Arabia, which is located in the west of the KSA, with almost four million and a half people living in the city (as of 2021). Jeddah is home to more than 40 primary healthcare centers that work to provide the best preventative and therapeutic services to patients, contribute to serving patients, and reduce pressure on the Ministry of Health (MOH) hospitals. Distributed according to its geographical division: north, south, east, and west. Each center belongs to an administrative and operational hospital affiliated with the MOH (25): King Fahd General Hospital, King Abdullah Medical Complex, East Jeddah General Hospital, Al-Thaghr Hospital, and King Abdulaziz Hospital.

Study population

The study population was hypertensive patients at the primary health care centers in Jeddah City, Saudi Arabia. All adult patients (age ≥18 years, female and male), with confirmed hypertension, treated with medications for at least three months, and with no cognitive and communicative disabilities were eligible for participation in this study. We excluded patients with cognitive impairments and language barriers from this study.

Sample size and sampling technique

Considering a 5% margin of error, a confidence level of 95%, 50% proportion, and using sample calculation software, Raosoft (http://www.raosoft.com/samplesize.html), the minimum sample size was 377.

A multistage cluster sampling technique was used to select eligible participants. Primary healthcare facilities were divided into five clusters according to the MOH hospitals they are affiliated with. Using a simple random sampling technique, two primary healthcare centers were selected from each cluster. The simple random sampling process was conducted using a software tool to randomly select the centers from the list to be included in the study. Finally, the number of participants from each center was chosen to be proportional to the number of patients served by each primary health care center selected.

Data collection tool (instrument) and procedures

An anonymous questionnaire was used for data collection and had five sections. Section I showed in detail the steps and aims of the study and ended with a question asking the respondents whether they agreed to participate. Section II inquired about the duration of the diagnosis and medication usage. Section III collected socio-demographic characteristics: sex, age, nationality, marital status, education, income, occupation, etc., and Section IV collected data on medication adherence among respondents. The Morisky, Green, and Levine (MGL) scale, originally developed in 1986 [22], was used for measuring medication adherence. The MGL four-item adherence scale was translated into the Arabic language with the intent of being cross-culturally adapted for the Arabic-speaking communities. As a consequence of the similar psychometric properties of the Arabic version and the original English version of the MGL scale, it has been found to be a reliable and valid scale. A comparison between the original Cronbach alpha of 0.61 and the new Cronbach alpha of 0.59 shows that the latter is comparable [23]. To measure medication adherence with this scoring scheme, each item is scored as "yes" = 1 and "no" = 0, with a total score ranging between 0 and 4, depending on the item. It is suggested that the sum of the number of "yes" answers indicates the level of non-adherence; a lower score indicates a higher level of adherence. The level of adherence among patients is classified as follows: high adherence for those scoring 0 ("yes" to no items), moderate adherence for those scoring 1-2 ("yes" to one or two items), and low adherence for those scoring 3-4 ("yes" to three or four items).

Section V used the Multidimensional Scale of Perceived Social Support (MSPSS) questionnaire to assess the perceived social support level. MSPSS is a 12-item questionnaire that assesses the perception of social support from three sources: family, friends, and a significant other, using a seven-point Likert scale [24]. The MSPSS scores are calculated by summarizing the responses across all 12 items for the total score or by taking an average for the mean score based on the responses. There are three subscales for scoring the significant other: the numbers 1, 2, 5, and 10 for the significant other, the numbers 3, 4, 8, and 11 for the family, and the numbers 6, 7, 9, and 12 for friends. Based on the scores, the support level is rated as low (1 to 2.9), moderate (3 to 5), or high (5.1 to 7) [24].

As part of the data collection process, the researcher scheduled visits to the administrator of each healthcare center and asked for permission. Then, using trained data collectors, they met the eligible patients, explained the purpose and objectives of the study, asked them to participate, and obtained informed consent. Finally, they handed out the questionnaire to the participants and started collecting data.

Data analysis

The data were analyzed using Statistical Package for Social Sciences (SPSS) version 27 (IBM Inc., Armonk, NY, US). The continuous variables were presented as mean and standard deviation while categorical variables were presented as frequency distribution and percentages. We used the chi-square test to assess the association between the outcomes (perceived social support level and medication adherence) and the independent variables (socio-demographic variables). An analysis of variance (analysis of variance (ANOVA) and post-hoc analysis were also used to determine the relationship between medication adherence and perceived social support. A confidence level of 95% was adopted throughout the study, and a P value less than 0.05 was considered statistically significant.

Ethical considerations

This study was approved by the Preventive Medicine Program Research Committee at the Public Health Department in Jeddah Governorate and the General Administration of Research and Studies in The Directorate of Health Affairs in Jeddah Governorate. We also obtained permission from the competent authorities of the Primary Health Care Centers included in the study. We obtain informed consent from the participants themselves before starting data collection. To ensure the anonymity of the study, no personally identifying information was collected, and all of the data collected were stored on a computer protected by a password to preserve the confidentiality of the study. The privacy of all participants was maintained throughout the study.

## Results

Socio-demographic characteristics

A total of 377 hypertensive patients were included in the study, with no missing data. The mean age of the participants was 57 years (SD = 10). The majority were male (n=226, 59.9%), and most were married (n=261, 69.2%). Education levels varied, with 148 (39.3%) having completed high school and 79 (21%) holding a bachelor’s degree or higher. The monthly income distribution showed that 166 (44%) earned less than 5,000 SAR while 47 (12.5%) earned more than 15,000 SAR. The occupational status ranged from government employees (n=121, 32.1%) to unemployed (n=29, 7.7%). Detailed socio-demographic characteristics are summarized in Table 1.

**Table 1:** Socio-demographic characteristics

Characteristics	Frequency (%)
Total	377 (100%)
Gender	
Male	226 (59.9%)
Female	151 (40.1%)
Age in years, mean (SD)	57 (10)
Marital status	
Married	261 (69.2%)
Widowed	47 (12.5%)
Divorced	36 (9.5%)
Single	33 (8.8%)
Education level	
Highschool	148 (39.3%)
Bachelor's degree or higher	79 (21%)
Intermediate	63 (16.7%)
Elementary	54 (14.3%)
Illiterate	33 (8.8%)
Monthly income, Saudi Arabian Riyals (SAR)	
SAR <5000	166 (44%)
SAR 5000-15000	164 (43.5%)
SAR >15000	47 (12.5%)
Occupation	
Government employee	121 (32.1%)
Retired	93 (24.7%)
Homemaker	67 (17.8%)
Private sector employee	67 (17.8%)
Unemployed	29 (7.7%)

Medication adherence

**Figure 1:**
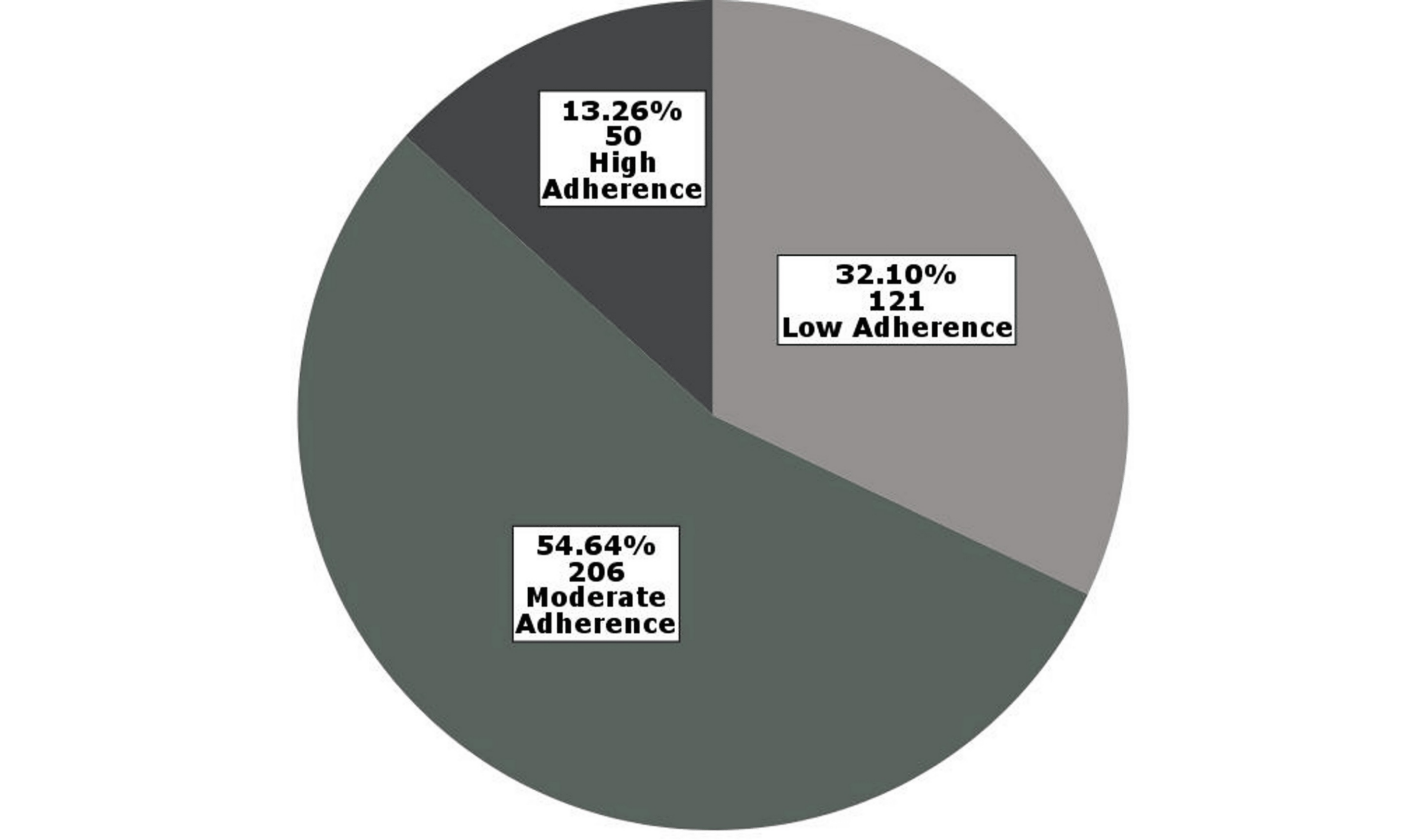
Distribution of levels of adherence to medications

**Figure 2:**
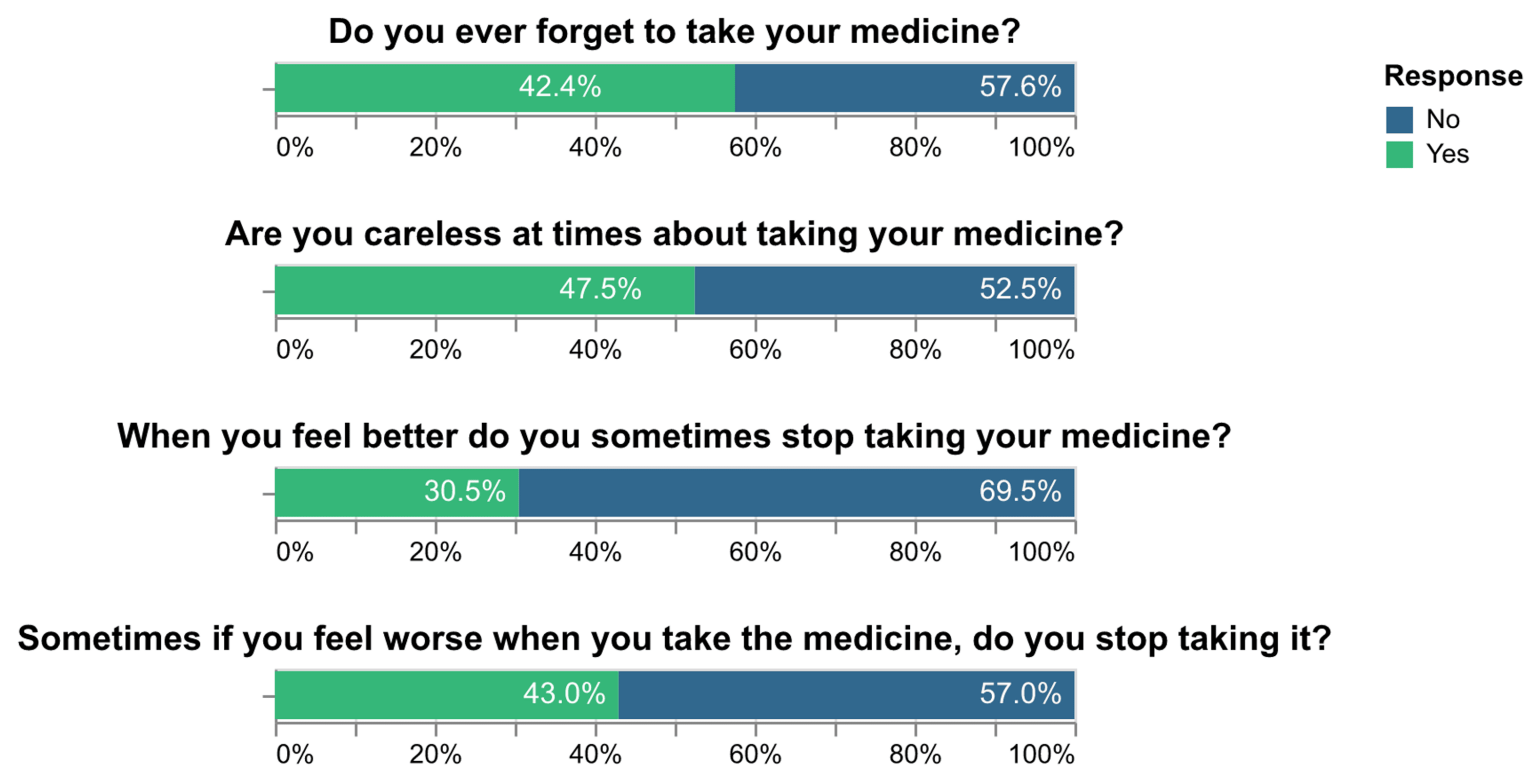
Distribution of responses to the MGL scale MGL: Morisky Green and Levine

Medication adherence levels, assessed using the MGL scale, showed that 121 (32.1%) participants had low adherence, 206 (54.6%) had moderate adherence, and 50 (13.3%) had high adherence (Figure 1). The distribution of responses to each question in the MGL questionnaire is presented in Figure 2.

Perceived social support

**Figure 3:**
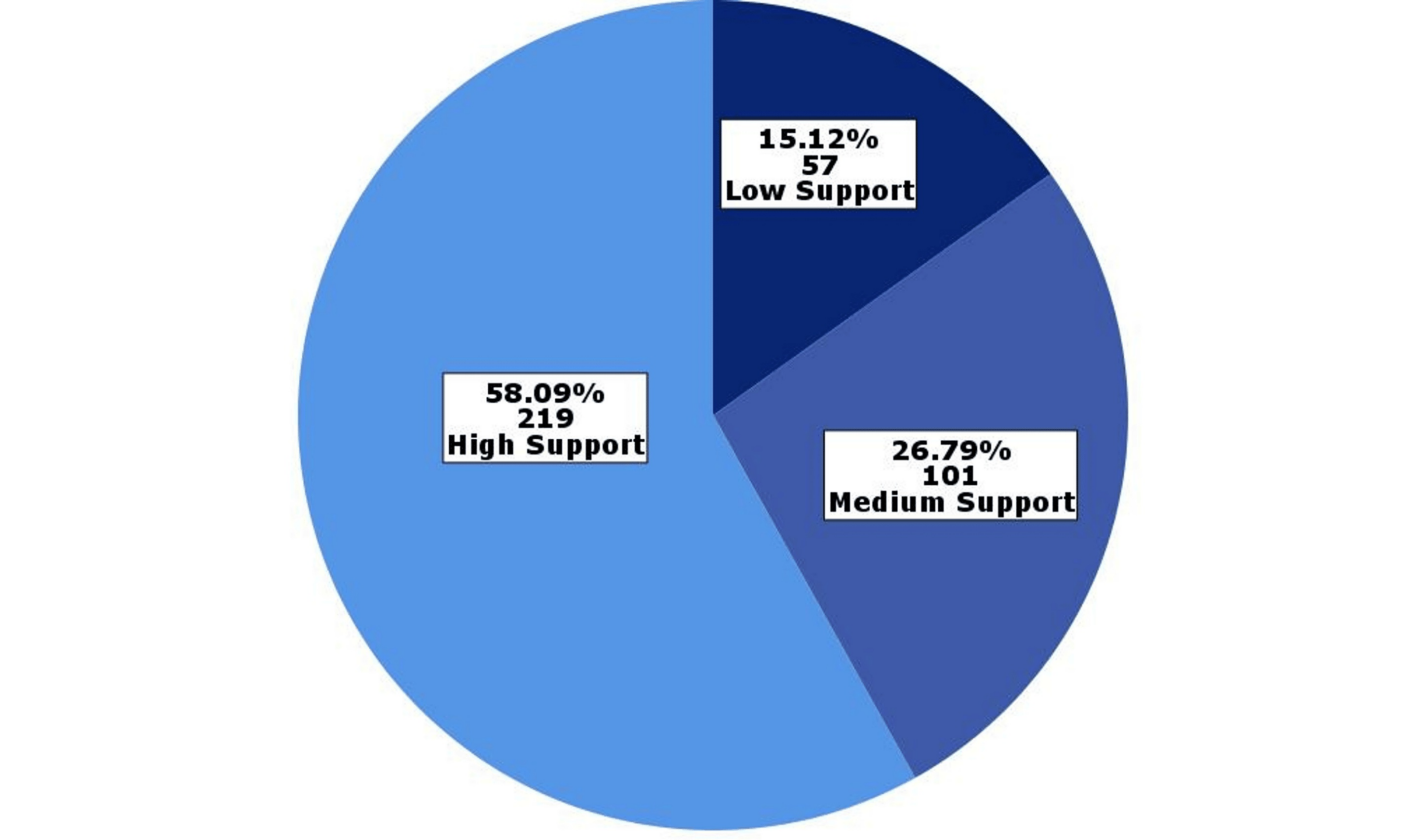
Distribution of levels of perceived social support

**Figure 4:**
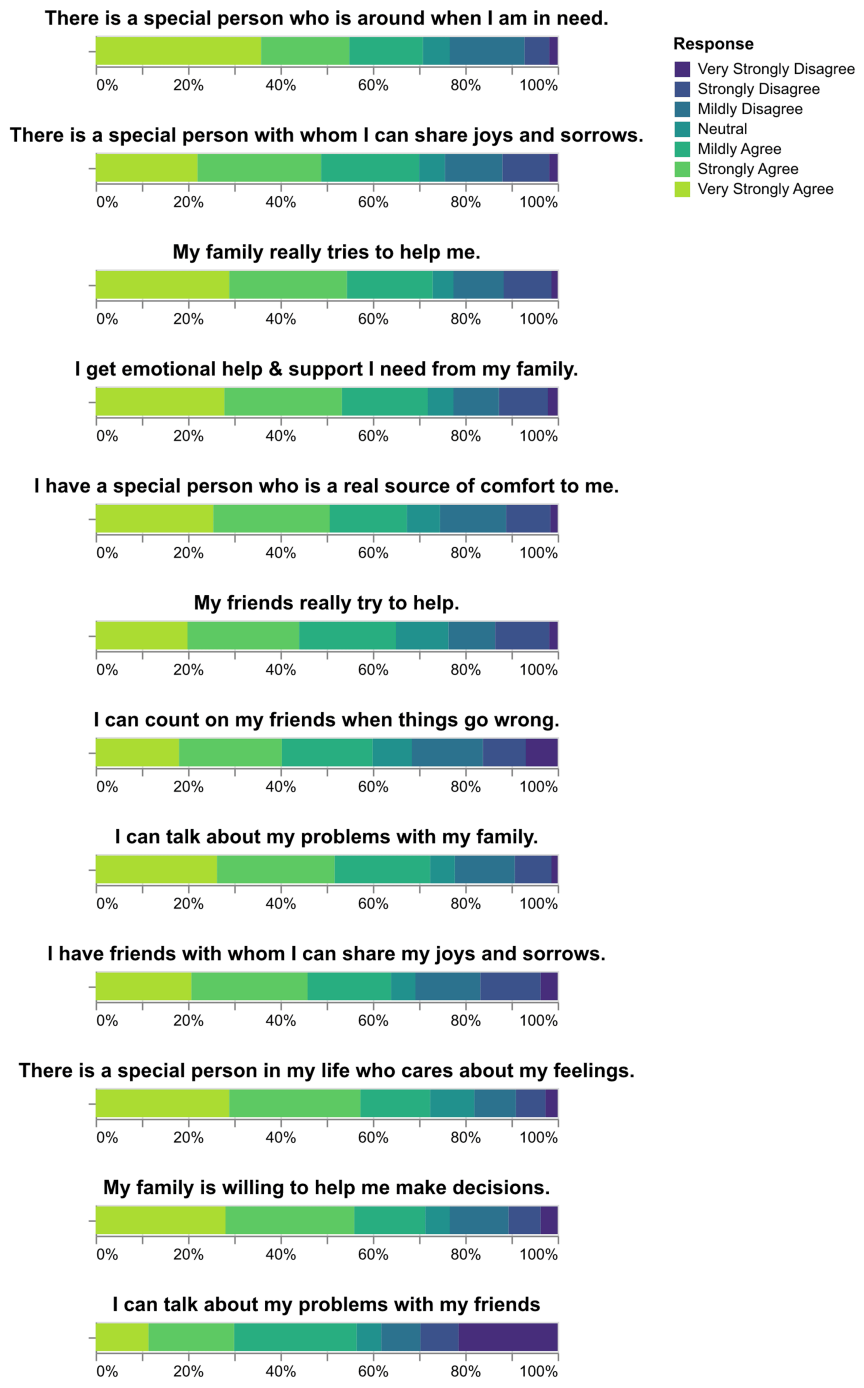
Distribution of responses to the MSPSS questionnaire MSPSS: Multidimensional Scale of Perceived Social Support

Perceived social support was measured using the MSPSS scale. The results showed that 219 (58.1%) participants reported high levels of social support, 101 (26.8%) reported moderate levels, and 57 (15.1%) reported low levels (Figure 3). The distribution of responses to each question of the MSPS questionnaire is presented in Figure 4.

Association between medication adherence and perceived social support

An analysis of variance revealed a significant relationship between medication adherence and perceived social support (F = 10.293, p < 0.01). Post hoc analysis indicated that participants with moderate adherence had significantly higher perceived social support scores compared to those with low adherence (Table 2).

**Table 2:** Mean MSPSS Scores Stratified by Levels of Adherence The ANOVA test was significant (F = 10.29, P<0.01); the Bonferroni test was used for post hoc comparisons MSPSS: Multidimensional Scale of Perceived Social Support; ANOVA: analysis of variance

Level of Adherence	Mean MSPSS Score (SD)	Significantly Different From
A. Low	4.52 (1.41)	B
B. Moderate	5.26 (1.37)	A
C. High	4.93 (1.59)	—

The chi-square test further confirmed a significant association between adherence levels and perceived social support categories (χ² = 25.292, df = 4, p < 0.01) (Table 3).

**Table 3:** Crosstabulation of levels of adherence and levels of perceived social support

	Level of Perceived Social Support		
Level of Adherence	Low	Medium	High
Low	23	48	50
Moderate	26	38	142
High	8	15	27

Influence of socio-demographic factors on medication adherence

The impact of socio-demographic factors on medication adherence was examined using chi-square tests, as loglinear analysis was not feasible due to the low number of cases in several categories (Table 4). Significant associations were found for marital status (χ² = 34.55, p < 0.01), education level (χ² = 15.57, p = 0.04), monthly income (χ² = 20.46, p < 0.01), and occupation (χ² =20.52, p < 0.01). Married participants were more likely to have moderate adherence while widowed participants had higher rates of low adherence. Higher education levels and monthly income were associated with greater adherence, with those holding a bachelor’s degree or higher and those earning over 15,000 SAR per month exhibiting the highest adherence levels. Government employees showed the highest rates of moderate adherence, whereas homemakers and unemployed participants were more likely to have low adherence. No statistically significant associations were found between gender (χ² = 0.174, p = 0.917) or age and levels of adherence.

**Table 4:** Levels of adherence stratified by socio-demographic factors

	Level of Adherence, Frequency (%)					
Factors	Low	Moderate	High	Total	χ2	P-value
Gender					0.174	0.917
Male	71 (31.4%)	124 (54.9%)	31 (13.7%)	226	—	—
Female	50 (33.1%)	82 (54.3%)	19 (12.6%)	151	—	—
Marital status					34.55	<0.01
Married	73 (28%)	159 (60.9%)	29 (11.1%)	261	—	—
Widowed	25 (53.2%)	18 (38.3%)	4 (8.5%)	47	—	—
Divorced	14 (38.9%)	18 (50%)	4 (11.1%)	36	—	—
Single	9 (27.3%)	11 (33.3%)	13 (39.4%)	33	—	—
Education level					15.57	0.04
Highschool	43 (29.1%)	90 (60.8%)	15 (10.1%)	148	—	—
Bachelor's degree or higher	24 (30.4%)	42 (53.2%)	13 (16.5%)	79	—	—
Intermediate	20 (31.7%)	31 (49.2%)	12 (19%)	63	—	—
Elementary	15 (27.8%)	32 (59.3%)	7 (13%)	54	—	—
Illiterate	19 (57.6%)	11 (33.3%)	3 (9.1%)	33	—	—
Monthly income, Saudi Arabian Riyals (SAR)					20.46	<0.01
SAR <5000	64 (38.6%)	87 (52.4%)	15 (9%)	166	—	—
SAR 5000-15000	47 (28.7%)	97 (59.1%)	20 (12.2%)	164	—	—
SAR >15000	10 (21.3%)	22 (46.8%)	15 (31.9%)	47	—	—
Occupation					20.52	<0.01
Government employee	27 (22.3%)	82 (67.8%)	12 (9.9%)	121	—	—
Retired	29 (31.2%)	51 (54.8%)	13 (14%)	93	—	—
Homemaker	30 (44.8%)	30 (44.8%)	7 (10.4%)	67	—	—
Private sector employee	23 (34.3%)	29 (43.3%)	15 (22.4%)	67	—	—
Unemployed	12 (41.4%)	14 (48.3%)	3 (10.3%)	29	—	—

## Discussion

The study explored the influence of perceived social support on medication adherence among hypertensive patients and provided valuable insights on how socio-demographic characteristics and perceived social support influence medication adherence among hypertensive patients in Jeddah, Saudi Arabia.

The findings show that participants who were married and had a higher level of education and wealth showed better adherence. This finding is consistent with prior research showing that higher education and income levels were associated with better medication adherence among hypertension patients [25]. Similarly, recent evidence indicated that marital status is a strong predictor of medication adherence, with married people being more likely to stick to their treatment plans due to enhanced social support [26]. Given the finding that married participants were more likely to have moderate adherence, spouse support may be an important component in increasing adherence. Spouses frequently serve as caregivers, assisting with medication management and providing emotional support. Spousal engagement has a favorable influence on the management of chronic illnesses such as hypertension, implying that treatments aimed at couples may enhance compliance [27].

The effect of occupation on medication adherence is consistent with earlier studies. For example, in one study, government employees showed higher adherence rates than homemakers and jobless participants [28]. This is consistent with the findings of another study that showed that employment gives structure and resources, leading to better health outcomes and adherence. Unemployed people, who may encounter economic difficulties and stress, tend to have lower adherence rates [29]. The lack of a significant relationship between gender and adherence in this study contradicts certain earlier findings. For example, women were found to be more likely than men to adhere to antihypertensive drugs, citing variations in health-seeking behavior [30,31]. This gap could be attributed to cultural and regional variables unique to Saudi Arabia, where the study was conducted.

The significant association between perceived social support and medication adherence supports prior research findings, underlining the importance of social support in chronic disease management. For example, studies showed that robust social support systems improve drug adherence across a variety of chronic conditions, including hypertension [15,32]. Social support from family, friends, and healthcare practitioners can help patients adhere to their treatment programs by offering emotional encouragement, practical assistance, and reminders. The current study's post-hoc analysis revealed that participants with moderate adherence scored significantly higher on perceived social support than those with low adherence. This shows that social support may act as a buffer for patients who struggle with adherence due to other factors such as socioeconomic status. This finding is reinforced by a previous meta-analysis that concluded that social ties have significant health advantages and can increase adherence by reducing stress and instilling a sense of accountability in patients [33]. In Saudi Arabia, strong family ties and religious duties may impact health practices. Cultural norms in the Middle East, such as community living and collective decision-making, can increase adherence to treatment or disease management when social support is viewed as a religious and familial responsibility [34,35]. This could explain why the study found such high levels of social support among those with moderate adherence. While family support was also a major factor in the perceived social support, it is crucial to investigate the influence of support from healthcare professionals and community networks. It was found that healthcare practitioners' assistance, including regular follow-ups and education, has a substantial impact on adherence [36]. This underlines the need for healthcare systems to develop strong patient-provider connections to support long-term adherence.

Given the strong association between social support and medication adherence, using digital health tools that enable social interactions may improve medication adherence. For example, Buis et al. found that mobile health interventions with social support components, such as reminders from family or friends, significantly enhanced adherence among hypertension patients [37]. In Saudi Arabia, where mobile phone use is strong, utilizing such technology may be an effective technique for improving adherence.

The study had some limitations for consideration. Its cross-sectional design limits the ability to establish causality, as it relies on self-reported data, which may be subject to recall or social desirability bias. The study's sample is drawn from primary healthcare centers in Jeddah, Saudi Arabia, and its findings may not be generalizable to other populations or regions due to cultural and healthcare system differences. The study's inclusion of willing participants may also introduce a sampling bias. Longitudinal data are needed to assess changes in medication adherence and social support over time. Other factors influencing medication adherence, such as psychological factors, medication side effects, and health literacy, were not explored. Therefore, we recommend extensive longitudinal studies to consider these limitations in order to give more comprehensive insights into perceived social support on medication adherence among hypertensive patients in Saudi Arabia.

## Conclusions

This study found that medication adherence among hypertensive patients is mostly moderate and found a significant relationship between perceived social support and adherence, with moderate adherence participants reporting higher levels of support. Socio-demographic factors, such as marital status, education, income, and occupation, also significantly influenced adherence levels. These findings are consistent with and build on earlier studies, emphasizing the relevance of social support systems, socio-demographic characteristics, and cultural influences in treating chronic diseases. Further studies should look into the integration of new technology-based interventions, such as digital health, and the role of healthcare providers in providing social support to increase adherence.
